# Research Trends of Human–Computer Interaction Studies in Construction Hazard Recognition: A Bibliometric Review

**DOI:** 10.3390/s21186172

**Published:** 2021-09-15

**Authors:** Jiaming Wang, Rui Cheng, Mei Liu, Pin-Chao Liao

**Affiliations:** 1 School of Economics and Management, Tongji University, Shanghai 200092, China; 1551288@tongji.edu.cn; 2 Department of Construction Management, Tsinghua University, Beijing 100084, China; chengr19@mails.tsinghua.edu.cn; 3 School of Urban Economics and Management, Beijing University of Civil Engineering and Architecture, Beijing 100084, China; liumei0311@126.com

**Keywords:** human-computer interaction, construction, hazard recognition, bibliometric review

## Abstract

Human–computer interaction, an interdisciplinary discipline, has become a frontier research topic in recent years. In the fourth industrial revolution, human–computer interaction has been increasingly applied to construction safety management, which has significantly promoted the progress of hazard recognition in the construction industry. However, limited scholars have yet systematically reviewed the development of human–computer interaction in construction hazard recognition. In this study, we analyzed 274 related papers published in ACM Digital Library, Web of Science, Google Scholar, and Scopus between 2000 and 2021 using bibliometric methods, systematically identified the research progress, key topics, and future research directions in this field, and proposed a research framework for human–computer interaction in construction hazard recognition (CHR-HCI). The results showed that, in the past 20 years, the application of human–computer interaction not only made significant contributions to the development of hazard recognition, but also generated a series of new research subjects, such as multimodal physiological data analysis in hazard recognition experiments, development of intuitive devices and sensors, and the human–computer interaction safety management platform based on big data. Future research modules include computer vision, computer simulation, virtual reality, and ergonomics. In this study, we drew a theoretical map reflecting the existing research results and the relationship between them, and provided suggestions for the future development of human–computer interaction in the field of hazard recognition from a practical perspective.

## 1. Introduction

According to the Encyclopedia Britannica, human–computer interaction is usually defined as “the science concerned with designing effective interaction between users and computers and the construction of interfaces that support this interaction” [1], i.e., the process of exchanging information between a human and a computer in a certain manner, using some kind of conversational language to accomplish a defined task [2]. As an interdisciplinary area of research involving many fields such as computer science, psychology, sociology, graphic design, and industrial design, human–computer interaction has evolved from the early stage of manual work to the stage of the web user interface and multimodal intelligent interaction, with user customization, embedded computing, augmented reality, social computing, knowledge-driven human–computer interaction, emotion interaction, and brain–computer interfaces as the focus of research [3], which has dramatically improved human life.

In the field of civil engineering, “hazard” is usually defined as “the source of energy that, if released and results in exposure, could cause injury or death” [4]. Due to the specificity of the construction industry, the overall hazard recognition rate for construction projects is relatively low compared with other industries, at 66.5%. At the individual level, in general, the recognition rate of hazards among construction workers with more than 10 years of experience is less than 80% [5]. Therefore, effective recognition of potential hazards is of great significance to reduce the accident rate in the construction industry and ensure the safety of construction workers. However, the traditional hazard recognition technology is single-modal and relies too much on humans’ subjective feelings. Consequently, the hazard recognition technology has progressed slowly and failed to meet the needs of construction industry development to date, which is one of the main reasons why the global number of casualties in the construction industry has still not clearly decreased [5]. For example, in the United States, the number of construction fatalities was 1102 in 2019, revealing an increase of 6.17% compared to 2018 [6]. Today, the effective recognition of potential hazards has become a major strategic issue related to the sustainable development of the construction industry, in addition to the safety of workers.

As a consequence, due to the pace of the fourth industrial revolution, human–computer interaction technologies are increasingly being applied to the construction industry, effectively driving advances in hazard recognition technologies. For instance, the modeling, measurement, and enhancement of the effectiveness of various types of interfaces between computer applications and construction workers, and maximization of the accuracy of the mapping of data from one modality to another, are academic frontiers that are currently being addressed by scholars [7]. Therefore, it can be inferred that the application of human–computer interaction technology in construction hazard recognition has a solid research foundation and a broad development prospect.

In this paper, we define CHR-HCI as the research related to both human–computer interaction and construction hazard recognition. Although some scholars have deeply explored this field, limited research has concentrated on providing a comprehensive overview for these studies. Thus, to systematically summarize the research related to this topic and identify the directions for future research, this study aimed to: (1) collect peer-reviewed papers and conference articles associated with CHR-HCI from 2000 to 2021 to ensure the literature’s completeness and representativeness; (2) extract the characteristics of the research in this field including the numbers, types, and country sources of articles by analyzing the basic information of the papers; (3) undertake keyword co-occurrence analysis and time-zone analysis to identify the research content and evolution trend; (4) summarize the research modules through cluster analysis and propose valuable potential research topics for scholars; (5) establish a research framework and a theoretical map of CHR-HCI to show the existing research progress and shortcomings in this field, and provide practical guidance and assistance for construction management.

The following sections of this paper are organized in accordance with the order of the bibliometric research. Section 2 discusses the research methodology and process, and Section 3 presents the basic information analysis. Next, the keyword co-occurrence network in Section 4 reveals the research content and the association between keywords over the past 20 years. Section 5 clusters the terms to present the research modules composed of keywords with a high association, and then the timeline in Section 6 analyzes the evolution of research themes and trends. Section 7 illustrates a theoretical framework for research in this field and provides an outlook on research topics to be further explored. Finally, the theoretical contributions, practical contributions, and limitations of this paper are summarized.

## 2. Research Process

### 2.1. Paper Retrieval

**Figure 1:**
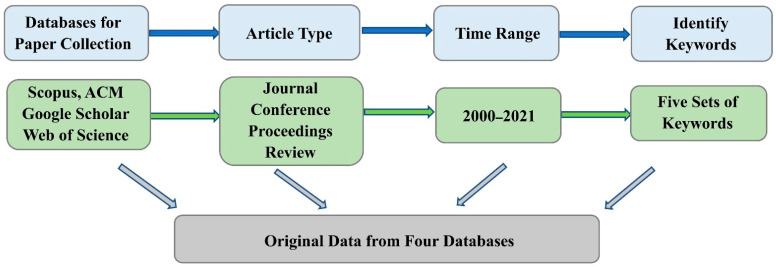
Paper retrieval process.

As shown in Figure 1 [8], the paper retrieval mainly included the following steps.

Firstly, we identified data sources. After careful consideration and repeated comparison, we selected Scopus, ACM Digital Library, Web of Science, and Google Scholar databases for the literature search. As important navigation tools, the four databases, which globally cover the most extensive abstracts, references, and indexes of academic literature, can help researchers understand the most advanced developments regarding CHR-HCI, thus explicitly identifying the current research topics and future trends.

Second, we selected the types of literature. The main literature resources of this study are journal papers about human–computer interaction technology and hazard recognition. In addition, because the research of hazard recognition and HCI depends on practical applications, conference papers should also be a necessary part of the literature resources, because academic conferences are an important channel for scholars to exchange research progress and solve scientific problems encountered in this field. Because these journal and conference papers have undergone strict peer review and selection before publication, the quality of these documents in the Scopus database is sufficiently high to represent the main body of the CHR-HCI research field.

Finally, we searched the literature based on the restrictions. The constraints of the paper retrieval mainly related to keywords and the time range. Regarding the time range, HCI, established in the 1950s [9], was first applied to hazard recognition considerably later. Thus, we selected 2000, when HCI was initially applied to the field of hazard recognition, as the starting year of the retrieval. In addition, before 2000, the quantity of relevant literature was minimal. In summary, we searched the papers published from 2000 to 2021. Regarding the keywords, we identified “construction”, “hazard”, “recognition”, “human-computer” and “interaction,” and searched the dictionary for synonyms and near-synonyms of each keyword. To ensure the completeness and comprehensiveness of the literature search, the following methods were used: Boolean operators were adopted to connect synonyms and near-synonyms, and the results were searched for in the databases. We added the synonyms and near-synonyms that had not been found previously, based on the keywords, abstracts, and papers with high relevance in the search results. After several iterations, if the number of documents we searched did not change significantly, we could determine the final search strategies. Taking Scopus as an example, the retrieval methods based on keywords and Boolean operators are shown in Table 1. When we used the other three databases to retrieve papers, we adjusted the Boolean operators according to the rules of each database.

### 2.2. Bibliometric Analysis Method

Human–computer interaction (HCI) is an emerging interdisciplinary subject, encompassing numerous fields, such as computer science, industrial design, psychology, behavioral science, organizational behavior, and physiology. After HCI was introduced into construction hazard recognition, many new research topics requiring multidisciplinary knowledge were spawned. As a result, due to the large number of academic papers, it is not feasible to conduct a manual analysis of such a complex interdisciplinary field. The large number of documents creates a tremendous workload, leading to the waste of substantial effort by researchers to identify the research focus and correctly classify the literature. Furthermore, literature classification based on researchers’ subjective interpretation is prone to human error, leading to a significant deviation between the conclusions drawn in the literature review and the facts.

Bibliometrics usually refers to the science that applies mathematical and statistical tools to quantitatively analyze literature resources and obtain relatively accurate and objective statistical results. Compared with the traditional literature classification method, bibliometric analysis can not only reduce the workload of researchers and shorten the research cycle, but also help researchers draw more scientific conclusions. As a consequence, the introduction of the bibliometric method is indispensable for the analysis of the literature in this field.

Bibliometric software is a fundamental tool for conducting bibliometric analysis. To explore the research progress of CHR-HCI and improve the efficiency of analysis, this study adopted a combination of CiteSpace and Vosviewer to undertake the bibliometric research. CiteSpace and Vosviewer, as typical bibliometric analysis software with an automatic calculation function, can be used by the researcher to visualize the theoretical rules, knowledge structure, and advanced content of the research field, and clearly reveal the relationship between two research topics to finally generate a visual chart called a “scientific knowledge map”.

Before using the software for bibliometric analysis, we needed to identify the final research sample. Because the aim of the paper search is to comprehensively retrieve relevant papers and avoid the exclusion of relevant articles, all of the literature obtained by the search may not be relevant to this study. Furthermore, the literature search will inevitably encounter the problem of duplicate research. For example, some papers are first accepted in conferences and then published in journals, thus generating duplicate publications. To address the above issues, the study sample was identified through the following three-level screening process.

First, the research team exported four datasets from four databases and then compared them by reading the titles carefully to identify an initial literature list that did not contain any apparent duplicates. Second, based on reading the publication titles, the literature abstracts were carefully checked, and thus irrelevant or duplicate papers were eliminated. Third, we browsed the general content of the literature and further eliminated the non-compliant literature on the basis of the first two steps. Finally, 274 eligible papers were selected as the study sample. Figure 1 shows the framework of the study methodology.

**Figure 2:**
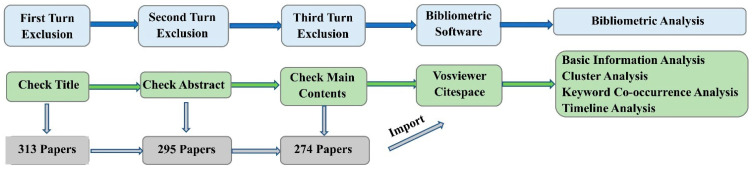
Bibliometric analysis method.

After identifying the sample, bibliometric analysis was conducted through CiteSpace and Vosviewer. Because the abstract and keywords are a powerful representation of the main idea of a paper, in this research we systematically identified the current research status and future development trends in this field using basic information analysis, cluster analysis, keyword co-occurrence analysis, and keyword timeline analysis. In addition, it should be noted that the corresponding indexes of cluster analysis should meet certain credibility criteria to obtain reliable research modules. Among the indicators describing credibility, the mean silhouette value P was used to measure the homogeneity of clusters, and modularity Q was used to represent the strength of connections between nodes. The results are usually considered to be reliable when the values of the two indicators are not lower than 0.7 and 0.3, respectively [10]. Figure 2 shows the process of bibliometric analysis [8].

## 3. Basic Information Analysis

After identifying the sample, we first analyzed the basic information of the 274 publications. Similar to the descriptive statistics in some experimental studies, the main purpose of this section is to help readers understand the basic information including the number of annual publications, the composition of literature types, and publication countries or regions in this field. Analysis of the number of annual publications aims to explore the trends of the literature, concentrating on CHR-HCI and its future development potential; the statistics relating to the literature types are used to reflect the research progress through the ratio of articles, conference papers, and reviews; and the analysis of publication countries/regions can reveal the global distribution of research and provide advice on location selection for scholars interested in this field.

### 3.1. Number of Annual Publications

**Figure 3:**
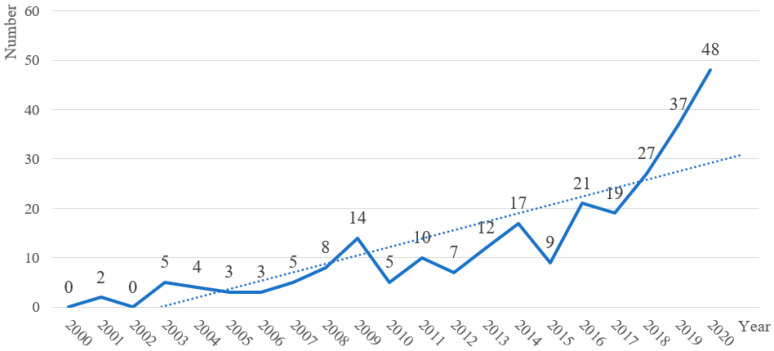
Distribution of related papers from 2000 to 2020.

The trend of annual publications in this field from 2000 to 2020 is shown in Figure 3. Before 2009, the number of relevant papers published in most years was small. Since 2011, particularly since 2015, publications have shown a significant upward trend, surging from nine papers in 2015 to 48 papers in 2020. Because the current year has not yet ended, the papers published in 2021 are not plotted in Figure 3 to avoid any visual misrepresentation for the readers. This figure shows that, despite the impact of the COVID-19 pandemic, the number of publications in this field is considerable.

Moreover, using the least-squares method, a regression model taking the number of publications as the dependent variable and the year as the independent variable was applied to reveal the trend of publications; the resulting slope is positive, as shown by the dashed line in Figure 3. In addition, we calculated the Price Index as the percentage of the number of publications in the last five years (defined as 2016–2020 because 2021 has not yet ended) divided by the total number of publications (defined as 2000–2020). The value of the Price Index was 0.594, showing that the literature in this field, rather than aging, has excellent research prospects. To conclude, the number of annual publications indicates that the research related to CHR-HCI has attracted a significant amount of attention and has been a burgeoning research area in recent years.

### 3.2. Composition of Literature Types

**Figure 4:**
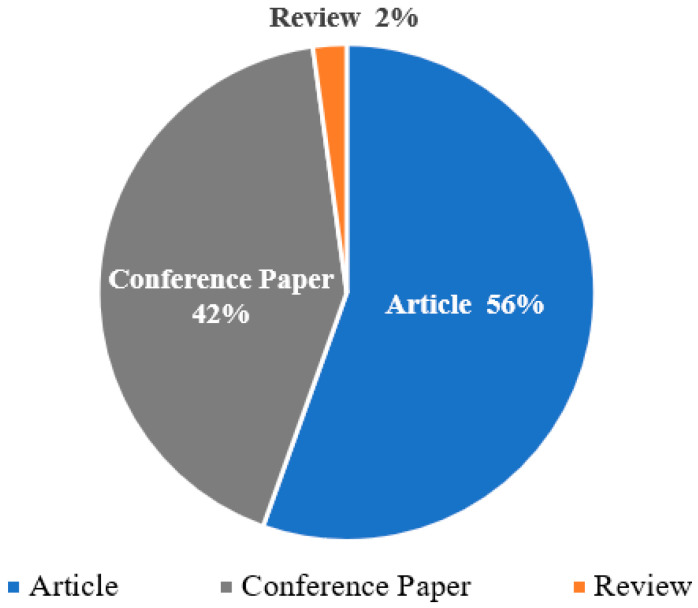
Distribution of literature types.

The types of the 274 documents are shown in Figure 4. The proportion of different literature types can reflect different research dimensions in the field. First, journal articles accounted for more than half of the articles, at 56%. Because journal articles usually represent basic research related to experiments, this reflects a certain amount of basic research in the field, which is still in a vigorous development stage. Second, conference papers also account for a large proportion, at 42%. The percentage of conference papers usually shows the excellence of the academic communication mechanism in this field; thus, the ratio of more than 40% proves that the area has a high research value and scholars have a strong will to communicate and improve together. Hence, more communication platforms have been established through academic conferences. Third, review articles are relatively scarce, accounting for only 2%, which indicates that the literature review has not kept pace with the rapidly emerging fundamental research, and further reveals that few scholars have systematically summarized the research in this field.

### 3.3. Publication Country/Region

**Figure 5:**
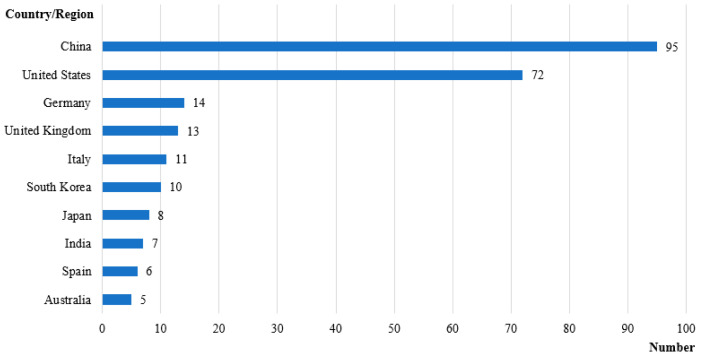
Publication country/region distribution.

As shown in Figure 5, 95 papers were published in this field in China and 72 in the United States, which is in line with the construction industry’s comprehensive strength and development level in both countries. As a country with an early start in the technological revolution, the USA has many advantages in developing emerging technologies, including a rich technology reserve, high-quality human resources, and good innovation performance. As the world’s largest market in the construction industry, China, which has numerous projects under construction, is able to provide researchers with rich practical opportunities. Thus, China has also made significant progress in the field of CHR-HCI. In addition, the UK, Germany, Korea, and Italy have also published more than 10 articles, showing that they are also essential contributors to the development of human–computer interaction applications in the construction industry.

In summary, we believe that future research in this field is expected to see greater cooperation among countries. At the national level, taking China and the United States as an example, China’s emerging infrastructure construction provides good opportunities for practice in the field of construction engineering safety. However, the rich technical reserves and high-quality human resources in the United States are also needed in China; thus, cooperation between the two countries can compensate for the disadvantages of each and allow the two to work together for a better future in hazard identification. At the personal level, scholars devoted to the field of hazard identification may consider going to UK, Germany, USA, China, or other suitable countries/regions to start research or actively cooperate with scholars in those countries/regions.

## 4. Keyword Co-Occurrence Network

The keyword co-occurrence network diagram shows, in detail, the centrality, importance, and connections of the research terms in a field. The keyword co-occurrence network consists of three basic elements: nodes, node markers, and links between nodes. The color of the node represents when the corresponding term was first explored. The cooler the node’s color, the earlier the node appeared. The radius of each node shows the frequency of the term, and the distance of its position from the network’s center represents its centrality. The width of the line between two nodes represents their connection strength, and the line’s color reveals the first time at which the two terms were first co-occurred [11]. Similarly, the cooler the link’s color, the earlier the two terms were connected.

**Figure 6:**
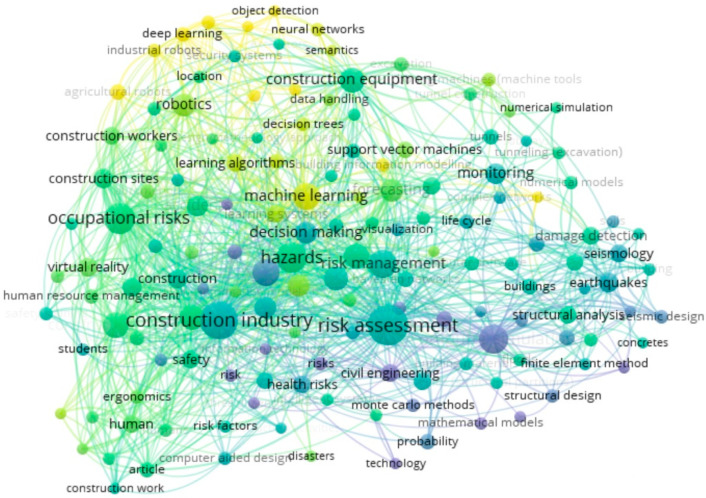
Keyword cooccurrence networks.

First, after keyword co-occurrence analysis was completed using Vosviewer, we obtained the scientific knowledge graph shown in Figure 6 [8], which identifies the research progress of CHR-HCI. Second, the mean silhouette (P) value obtained after cluster analysis of the keyword co-occurrence network is 0.7533 and the modularity (Q) is 0.796, both of which meet the corresponding credibility criteria. Consequently, we can directly adopt the keyword co-occurrence network shown in Figure 6 to make clustering analysis. Finally, the research terms in Figure 6 can be divided into three categories according to the levels they belong to. The first level is the research goal, i.e., keywords related to construction safety and hazard recognition; the second level is the general method to achieve the goal, i.e., keywords related to human–computer interaction; and the third level is the specific technology adopted by researchers, i.e., keywords related to machine learning and specific algorithms.

### 4.1. Terms Related to Construction Safety and Hazard Recognition

In the past 20 years, an important shift has occurred in the research field of CHR-HCI, i.e., the guiding ideology has changed from post-accident analysis to accident prevention and hazard prediction [12]. The term “forecasting” in Figure 6 reveals this important shift. In contrast to post-accident analysis, accident prevention and hazard prediction emphasize that construction-related staff should accurately identify potential risks and take appropriate measures to avoid accidents before an accident occurs. The emergence of key words such as “risk perception” and “risk analysis” is closely related to the change in this guiding ideology, in that effective risk perception and analysis are essential for staff to accurately recognize hazards in complex construction environments [13].

Furthermore, in the field of risk prediction and hazard recognition, research on earthquakes, a major natural hazard, have attracted the continuous attention of scientists. Scholars have launched useful explorations from the perspectives of seismic design, building planning, and new materials [14]. Terms such as “earthquakes”, “seismic design”, “seismology”, “architectural design”, and “reinforced concrete” reflect the research progress of this subject, whose main research directions include earthquake prediction, real-time intelligent monitoring of earthquakes, design of new seismic structures, and development of new seismic resistant materials [15].

The keyword co-occurrence network also reflects the changes in the organizational management in this field. A revolution in organizational management and safety technologies is required to ensure the purpose of accident prevention and hazard prediction becomes reality [16]. Because construction safety is closely connected to organizational management, researchers are continuously hoping to improve safety performance through innovations in management. Terms such as “decision making”, “monitoring”, “safety training”, and “risk management” are vivid reflections of the changes in this field. In the past 20 years, the concepts of risk management, risk decision making, engineering structural health, and safety training in engineering construction have been generally accepted and have become important research topics of current scholars [17].

### 4.2. Terms Related to Human-Computer Interaction

In the practice of construction safety, it is not enough to merely rely on correct concepts and high-quality management to make breakthroughs in improving the hazard recognition rate—it is imperative to make changes in safety technology, which has been significantly improved by the introduction of human–computer interaction technology.

Human–computer interaction, usually occurring in the implementation of a specific automated task, refers to a mode in which humans and computer-related equipment share an operating space [18]. It has triggered a significant technological change in hazard recognition. Current HCI research in hazard recognition can be divided into three main categories: key technologies, typical products, and product performance.

Key technologies refer to basic or breakthrough technologies needed in the formation of HCI-related products applied to hazard recognition. Basic technologies generally include sensor technology, positioning and map construction technology, robot operating systems, 3D modeling technology and virtual simulation technology [19], whereas breakthrough technologies refer to computer vision, computer simulation, neural network, and high-performance material manufacturing technology [20]. As shown in Figure 6, terms such as “virtual reality”, “three-dimensional computer graphics”, “computer simulation”, and “computer vision” reflect the researchers’ focus on technology.

To date, researchers have developed typical products for HCI with certain hazard recognition functions, mainly including construction robots for specific scenarios and automated construction systems for integrated scenarios. Robots used for a single scene are capable of repeatedly completing specified tasks, such as excavation robots, handling robots, and painting robots that can complete hazard recognition in a specific scene [21]. Automated construction systems used for integrated scenarios usually have the ability to integrate multiple single-task robots, such as ABCS systems and SMART systems with more complete hazard recognition functions [22].

Product performance of HCI applied to hazard recognition refers to the product attributes, product cost, operation efficiency, operation quality, operation safety, etc. The performance can be evaluated either by horizontal comparison of typical HCI products and traditional operation methods in terms of product cost, operation efficiency, operation quality, and operation safety, or by vertical comparison of different HCI products in terms of human resources, building material consumption, machine quality, machine power, machine load, movement speed, operation accuracy, etc. [23]. In the future, HCI products applied to hazard recognition are expected to pay more attention to the directions of integrating design and construction, improving the mobility of humanoid robots, and enhancing the load capacity and the positioning accuracy of intelligent machinery [24].

### 4.3. Terms Related to Machine Learning and Specific Algorithms

Machine learning is the foundation of artificial intelligence, and deep learning is the further development and extension of machine learning. Compared with traditional neural networks, deep learning sets multiple implicit layers and thus has higher training efficiency and a better learning effect in the fields of computer vision, audio recognition, and natural language processing [25]. Under the CHR-HCI framework, deep learning is mainly used for data processing in the development experiments of HCI devices, writing of internal programs for electronic HCI devices, and risk prediction for hazard recognition systems [26]. To further explore deep learning, researchers are focusing on algorithms such as convolutional neural networks, stacked autoencoder network models, deep belief networks, etc. [27].

As shown in Figure 6, among the algorithms related to machine learning, the support vector machine (SVM) is widely applied to the research and development of devices related to hazard recognition. SVM, proposed by Soviet scientists in 1964, is a generalized linear classifier for binary classification of data using the kernel method in a supervised learning manner [28]. Since the 1990s, SVM has been rapidly popularized with the breakthrough of HCI-related technology, and thus a series of improved and extended algorithms have been derived. Because SVM can solve pattern recognition problems, such as portrait recognition, action recognition, emotion recognition, text classification, handwritten character recognition, etc., it has also been applied to hazard recognition. Recently, the improved algorithms of SVM applied to hazard recognition have encompassed improved algorithms for skewed data, probabilistic SVM, multiclassification SVM, least-squares SVM, structured SVM, and multi-kernel SVM. The extended algorithms include support vector regression, support vector clustering, and semi-supervised SVM [29]. As an algorithm still in the optimization phase, there are three main future research directions for SVM: the improvement of kernel functions, classification of big data, and combination of models [30].

As a multi-disciplinary subject, machine learning also involves multiple disciplines such as probability theory, statistics, approximation theory, convex analysis, and algorithmic complexity theory. Keywords such as “numerical/mathematical models” and “Monte Carlo methods” show the importance of numerical simulations represented by Monte Carlo simulations. Depending on the computer technology, the modern Monte Carlo simulation has two main advantages, i.e., simplicity and speed, and has become an important technical basis in construction project management [31]. Monte Carlo simulation is now an important tool for computer simulations in hazard recognition experiments.

## 5. Cluster Analysis

Because keyword co-occurrence networks are too detailed to simplify research subjects and identify research modules, we used cluster analysis to summarize the main trends of CHR-HCI.

Cluster analysis is an analysis mode in which text data are processed with optimized computational methods in statistics to obtain potential research themes. In this study, a combination of Vosviewer and CiteSpace was selected for cluster analysis, i.e., CiteSpace was used to optimize Vosviewer’s cluster analysis results. In CiteSpace, there are three usual methods for determining module names: log-likelihood ratio, mutual information, and highest word frequency [32]. Due to the representativeness of the module names, we chose the highest word frequency method to identify the modules.

**Figure 7:**
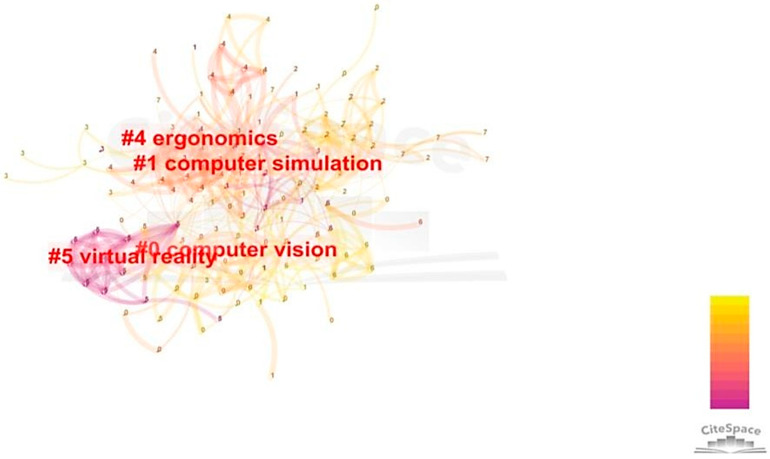
Cluster analysis.

After analysis and optimization, we obtained four modules with no obvious containment relationship, as shown in Figure 7 [8]: computer vision, ergonomics, computer simulation, and virtual reality. Because the keywords are not clearly shown in this figure, we listed the top three most frequently occurring keywords in each cluster (except the cluster label) in Table 2.

### 5.1. Cluster 1: Computer Vision

Among the 251 articles retrieved, 177 articles were related to this keyword, indicating that computer vision played a pivotal role in hazard recognition experiments. Advances in computer vision technology are based on the continuous optimization of deep learning algorithms, such as convolutional neural networks, stacked autoencoder network models, and deep belief networks [33,34]. The vital research topics include content-based image extraction, pose evaluation, multimodal data recognition, autosomal motion, image tracking, scene reconstruction, image recovery, and system integration [35].

In the field of hazard recognition, computer vision is divided into two research themes.

One is the development of models. The algorithms related to computer vision are based on the cognitive psychological models represented by the template matching model and the recognition-by-component theory model [36]. However, the algorithms mentioned above can only distinguish the low-visual-complexity hazard from a fixed perspective, which is challenging to adapt to the dynamic construction scenes with time-variant characteristics. In recent years, feature matching models have also been proposed to take into account the dynamic nature of the temporal dimension [37], but due to the lack of sufficient data to train the models and the inaccuracy of inter-modal mapping, the effectiveness of hazard recognition remains unreliable.

The other is the analysis of cognitive associations. Driven by recognition, human attention can be differentially distributed into different regions and shifted over time. Computers lack this ability to exploit and learn human attentional cues, which is one of the important factors restricting the development of computer vision techniques [38]. Moreover, the existing computer vision’s hazard judgment logic is based only on real-time construction scenarios and cannot combine existing cues to make predictions and inferences about future safety status. Therefore, future researchers should not ignore the time-variant nature of visual behavior, but are expected to focus on the logical association between existing cues and determine potential hazards.

### 5.2. Cluster 2: Ergonomics

Since 2015, ergonomics has become closely associated with CHR-HCI, and has developed in the directions of diversification, humanization, and intelligence. Currently, researchers are trying to use physiological and psychometric means to study the rational coordination relationship between the structural-functional, psychological, and mechanical aspects of the human body and computers, which aims at improving the performance of hazard recognition [39]. Sixty-five of the 251 articles retrieved were connected with this keyword, confirming that the interaction between construction hazard recognition and ergonomics is adequate and a large number of researchers have deeply explored the technical means. At present, research in this field focuses on task evaluation and quantification, brain-computer interfaces, and experimental paradigms in engineering psychology [40].

Akanmu et al. described a cyber-physical postural training environment where workers could perform work with reduced ergonomic risks [41]. Inyang et al. illustrated a proposed methodology to assess and quantify the ergonomic hazard effects of wall framing tasks on the back, legs, neck, shoulder, hands, and wrists of residential construction workers involved in manufacturing wood framing activities in a construction factory [42]. The focus was placed on risks associated with awkward work postures, force and static loading, contact stress, hand-arm vibration, repetitive tasks, and environmental factors. In addition, the contributions of organizational factors and the total daily duration of exposure to each risk factor were presented. The proposed methodology has been being incorporated into a computer program, “ErgoCheck”, which has shown success in quantifying work-related ergonomic hazards.

### 5.3. Cluster 3: Computer Simulation

Computer simulation, also known as computer emulation, is a computer program used to simulate an abstract model of a specific system [43]. Ninety-seven of the 251 articles retrieved were related to this keyword. Currently, computer simulation research related to hazard recognition is oriented towards discrete simulation, analogous simulation, simulation-based on probe elements, and simulation of stochastic processes or deterministic models, with the primary purpose of simulating hazards in construction scenarios through simulation software and external parameters. This research involves the development of source code and the optimization of existing programs [44]. Numerous scholars have continuously optimized discrete event simulation languages, such as GPSS, SIMSCRIPT, GASD, CSL, and SIMULA, in addition to continuous system simulation languages represented by DARE, ACSL, CSS, and CSSL, which have laid a solid foundation for human–computer interaction technology and promoted the development of the field of hazard recognition [45].

### 5.4. Cluster 4: Virtual Reality

Virtual reality (VR) technology is a human–computer interaction system with the function of creating a virtual world, which can immerse the users in the virtual environment [3]. Among the 251 articles retrieved, 52 were related to this keyword, indicating that virtual reality has a broad development prospect after being introduced into the field of hazard recognition. From the perspective of technology development, scholars are working on optimizing four key technologies: dynamic environment modeling technology, real-time 3D graphics generation technology, stereo display and sensor technology, and system integration technology [46]. From the perspective of technology application, virtual reality technology is mainly developed for safety training of construction workers and building risk assessment systems [47]. From the standpoint of technical disadvantages, the main problems faced by virtual reality include the high cost of production and unstable user visual experience [45].

For example, Huang et al. noted that a virtual reality system composed of a brain–computer interface (BCI) and electroencephalogram (EEG) neural network was constructed to collect users’ EEG signals and evaluate the accident susceptibility of construction workers. Based on the EEG and physiology data, a statistical model is used in the safety assessment framework to establish the risk standard. In addition, augmented reality has also been used to provide safety training for workers by superimposing virtual scenes onto the real world, thus enhancing the visual experience and reducing the production expense [48,49]. Therefore, using augmented reality to circumvent some of the drawbacks of virtual reality technology will also be a meaningful research topic in the field of CHR-HCI in the future.

## 6. Keyword Timeline Analysis

For this section, we undertook keyword timeline analysis to obtain a dynamic graph reflecting the evolution of popular research topics. The timeline analysis diagram consists of three elements: nodes, node markers, and lines between nodes. Each element in this graph represents the same meaning as that in the keyword co-occurrence network graph, except that this graph shows the year in which each keyword appears via a timeline [50].

**Figure 8:**
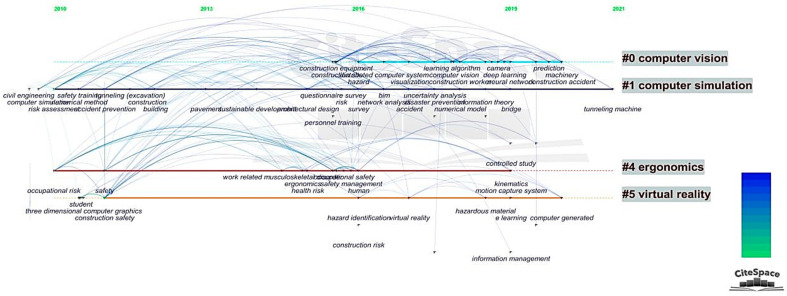
Timeline analysis.

The results of the timeline analysis are shown in Figure 8. Because the small number of publications prior to 2010 resulted in a blank between 2000 and 2010 in the final analyzed image, Figure 8 mainly shows the research dynamics between 2010 and 2021 [8]. The portion of literature published from 2000 to 2010, as a percentage of the total literature, is 17.88%. After reading and analyzing the papers, we found that the keywords that appeared relatively frequently in the research during this period were “computer simulation”, “project management”, “civil engineering”, etc. At that time, the computer simulation technology used for hazard recognition was not mature, and the discussion of hazard recognition in project management was not detailed, indicating that the research on CHR-HCI was in an initial stage during this period. Although the research published between 2000 and 2010 was relatively superficial, it provided a solid foundation for subsequent developments. Additionally, this study categorized the keywords in the timeline analysis based on the cluster analysis results, aiming at presenting the most valuable conclusions to readers.

From 2010 to 2011, terms such as occupational risk, construction safety, accident prevention, and risk assessment emerged, confirming our judgment in Section 4.1 that hazard recognition and construction safety gained researchers’ attention, and the opinion of accident prevention was gradually established in academic circles. Moreover, at this time, computer simulation, safety training, and 3D computer graphics were also initially applied to the study of hazard recognition to undertake accident prevention and risk prediction.

In 2013, terms such as sustainable development became a popular research topic, which symbolized the further maturation of guiding ideas in construction hazard recognition. Researchers combined the goal of achieving accident prevention with the new concept of green, healthy, safe, and sustainable development, which has been commonly advocated globally, to drive progress in hazard recognition. Moreover, terms such as “pavement” also emerged in 2013, which was mainly because the HCI research in intelligent driving could consistently provide meaningful references for hazard recognition in the construction industry: improving the design of the interaction interface including buttons, lights, and displays; setting up enclosures to divide the operating space between humans and computers; developing convenient mode-switching buttons to quickly start, change, and stop tasks; placing light curtains, laser sensors, and pressure-sensing mats to reduce the risk of irrelevant personnel entering the workspace unintentionally [51].

The year of 2016 was a landmark during which keywords containing safety management, health monitoring, construction equipment, virtual reality, construction informatics, and ergonomics emerged and established strong links with the preceding keywords of occupational risks, construction safety, and accident prevention. This progress reveals that, subsequent to the accumulation of the previous 15 years, an increasing number of scholars engaged in this research and published numerous research results in this year. Research at this stage focused on studying methods and models, mainly including safety training based on individual characteristics, questionnaire surveys, work-related musculoskeletal system monitoring, and wearable devices and sensors [52].

During 2018–2020, research on numerical models, deep learning algorithms, uncertainty analysis and neural networks became popular. These keywords representing specific algorithms and analysis methods mainly address computer vision and computer simulation, which is an inevitable continuation of the research growth that occurred around 2016. Many results emerged in this phase, such as motion capture systems, e-learning, information integration management systems, and improved support vector machines, representing a further deepening of algorithmic research in the field of CHR-HCI [53].

## 7. Framework Development, Future Directions and Discussion

### 7.1. Framework Development

**Figure 9:**
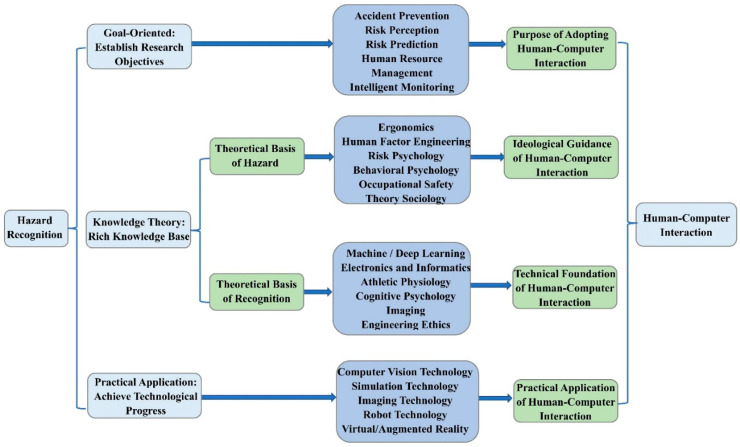
Research framework: from hazard recognition to HCI.

**Figure 10:**
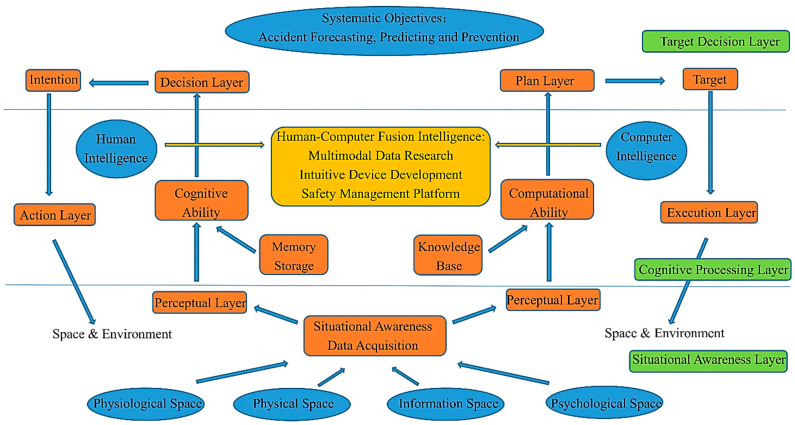
Hazard recognition based on human–computer fusion intelligence.

To reflect the overview of the research field from 2000 to 2021, we mapped the research framework and knowledge structure of CHR-HCI based on bibliometric analysis, as shown in Figure 9 and Figure 10 [8,36].

Because human–computer interaction is an emerging interdisciplinary field encompassing various fields, and hazard recognition also involves complex theoretical knowledge and practical techniques, a new framework was proposed in this paper to divide the CHR-HCI field into three levels:

(I) In terms of goal orientation, hazard recognition aims to study the process of identifying, perceiving, and recognizing hazards, and their influencing factors, in the construction environment for the purpose of risk perception, accident avoidance, potential hazard prevention, accident prediction, and intelligent monitoring. The change from post-accident analysis to pre-accident prediction and prevention is the core advancement in engineering safety guiding ideology within the last 20 years with the development of human–computer interaction technology. Therefore, this is also the aim of introducing HCI technology.

(II) From the aspect of theoretical foundation, hazard recognition mainly contains two parts: hazard-related/risk-related theory and recognition-related/identification-related theory. Engineering hazard-related theories include risk psychology, ergonomics, human factors engineering, behavioral psychology, sociology, and other disciplines [54], which are theoretical guides for the application of HCI technology. For example, the concepts of people-oriented and sustainable development in ergonomics provide theoretical directions for HCI technology. Recognition-related theories, as the technical basis of conducting HCI-related studies, mainly cover cognitive psychology, machine learning, deep learning, athletic physiology, imaging, electronics, informatics, etc. [55]. Furthermore, in addition to the development of science and technology, engineering ethics has become essential, and its supervisory role in scientific experiments has attracted significant attention from academic circles [56]. Thus, engineering ethics should also be regarded as a crucial guiding theory for hazard recognition.

(III) Regarding practical applications, the practical application of hazard recognition should focus on computer simulation technology, computer vision technology, virtual reality technology, augmented reality technology, and robotics [57]. We believe the emphasis of future research on hazard recognition should be placed on three issues. The primary goal of researchers is to explore appropriate methods to process multimodal data in hazard recognition experiments, and then to develop intuitive devices for hazard recognition using the appropriate data processing methods, as noted above. The ultimate goal is to establish an integrated safety management platform based on the development of suitable multimodal data processing methods and intuitive devices. Therefore, these three research aspects have not only been applied to a certain extent, but also represent explicit directions for the future.

In summary, as shown in Figure 9, we can construct the relationship between hazard recognition and human–computer interaction. Starting from the three dimensions of hazard recognition, namely, goal orientation, theoretical basis, and practical application, we can divide the CHR-HCI research field into three aspects, among which the theoretical basis can be further classified into the two aspects of hazard-related theory and recognition-related theory. These aspects eventually support human–computer interaction, illustrating the close and complex relationship between hazard recognition and human–computer interaction.

As a static schematic diagram, although Figure 9 provides a clear description of the research framework in this area, to better illustrate the implications of Figure 9 from a dynamic perspective, we drew Figure 10 to show the framework of hazard recognition from the perspective of human–computer fusion intelligence. The system consists of three layers: the situational awareness layer, cognitive processing layer, and target decision layer [36].

(I) The situational awareness layer is mainly used to collect information. In general, there are three elements in human–computer interaction systems: humans, computers, and environments. The humans provide the physiological and psychological space, the computers offer the information space, and the environments afford the physical space [36]. Through situational awareness, human–computer fusion intelligence can collect information from the above four kinds of spaces and import it into the perception layer of the humans and computers. The design of the perception layer requires the use of technologies such as wearable devices, combined with the disciplines of athletic physiology, imaging, electronics, and informatics, as shown in Figure 9.

(II) The cognitive processing layer, between the situational awareness layer and target decision layer, has the function of analyzing the data obtained from the perception layer by fusing the intelligence of the human and computer. Hazard recognition with human–computer fusion intelligence is expected to enable the processing of multimodal data, the development of intuitive devices, and the construction of integrated safety management platforms, which echoes the practical applications in Figure 9. Humans have the cognitive ability to analyze data combined with memory storage, whereas computers have the computational capability to process data in combination with a knowledge base. As a result, human–computer fusion intelligence has both the superb computing power of computers and the intelligent cognitive ability of humans, thus forming a dynamic closed loop of “perception”—“analysis”—“decision”—“execution”—“feedback”, which continuously optimizes the process of hazard recognition. In this process, cognitive and computational analysis corresponds to machine learning and deep learning in Figure 9.

(III) The target decision layer is able to make judgements subject to data analysis. Through cognition, humans can make decisions and form intentions, leading to corresponding actions. Similarly, through computation, computers can complete planning and determine goals, leading to the execution of feedback [36]. The systematic goals in Figure 10, also highlighting accident prediction and prevention, are consistent with the goal orientation in Figure 9, and the process of making decisions is closely related to the risk psychology, ergonomics, human factor engineering, behavioral psychology, and sociology in Figure 9.

**Figure 11:**
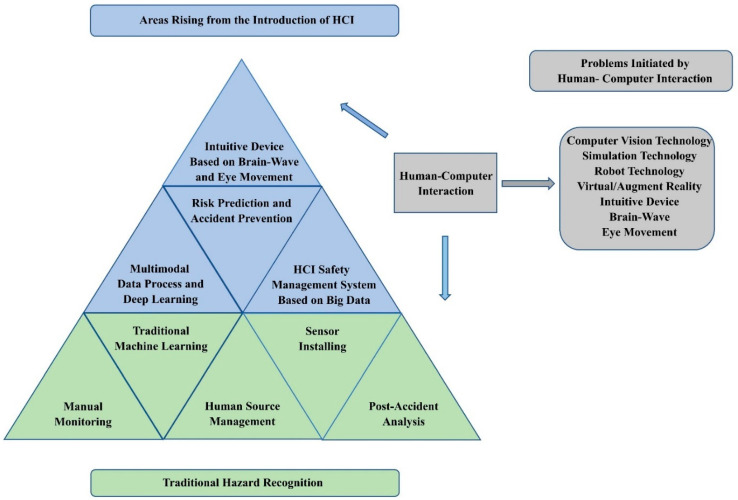
Interaction of hazard recognition and HCI.

In addition, to further explain the framework in Figure 9 and Figure 10, we list the traditional research areas of hazard recognition and the new research subjects arising from the introduction of HCI in Figure 11 [8]. Traditional hazard recognition mainly depends on manual monitoring, traditional human source management, and post-accident analysis, whereas current hazard recognition concentrates on risk prediction, accident prevention, deep learning, intuitive device based on brain-wave and eye movement, multimodal data process, etc.; thus, the subjects initiated by human–computer interaction relate to virtual reality, augmented reality, computer vision, computer simulation, etc. After identifying the framework of CHR-HCI, we are able to define the direction of future research.

### 7.2. Future Directions

As mentioned above, we summarized three main directions with recursive relationships: improving algorithms to process multimodal data, developing intuitive devices for hazard recognition, and building integrated safety management platforms.

#### 7.2.1. Improve Algorithms to Process Multimodal Data in Hazard Recognition Experiments

To effectively recognize hazards, a large number of studies have monitored workers’ physiological data in construction scenarios and explored the relationship between physiological data and hazard recognition performance. The application of multimodal methods has energized the research on hazard recognition, overcome the restrictions caused by unimodal data, and generated a series of new research topics following the combination with human–computer interaction technology.

(I) Multimodal representation learning. The data obtained from hazard recognition experiments generally have more than three modalities; however, current specific representation learning is limited to the case of two modalities. Consequently, it is recommended that researchers expand the number of modalities to which specific representation learning can apply. Furthermore, because dynamic real-time monitoring is an important future direction for hazard recognition experiments, and the mainstream methods of representation learning are often confined to static conditions, identifying approaches for the use of multimodal physiological data for dynamic deep learning is also a frontier issue [58].

(II) Translation among modalities. Because the physiological signals obtained from the hazard recognition experiments are of various types, including brain waves, visual pathways, heart rate, blood pressure, respiratory rate, speech, audio, and video, translation among modalities is needed in the studies. However, there is usually no single correct answer or optimal solution in this process, and the subjective consciousness of researchers can also have a large impact on the results; thus, the final results of hazard recognition experiments often fail to confirm the representation of the same entity between different modalities. It remains a challenge for a series of evaluation systems proposed by academia, such as BLEU, ROUGE, Meteor, and CIDEr, to address the subjectivity problem of the results [59].

(III) Multimodal semantic alignment. In the study of hazard recognition, multimodal semantic alignment is an important step in the analysis the data, and the problems in this field are mainly as follows. First, semantic alignment faces the problem of temporal effects because most data in hazard recognition experiments have temporal relationships. Next, it is difficult to design indicators to measure the similarities between different modalities, and manual design is time consuming and laborious. Furthermore, because experimental data may be missing or redundant, elements in different models may have one-to-many or many-to-one relationships, or may not be able to be matched if significant data are missing. When the elements are incorrectly matched, the performance of the model will be seriously degraded [60]. Finally, the performance of multimodal semantic alignment can be significantly influenced by noise.

(IV) Multimodal data fusion. Similar to multimodal semantic alignment, noise also affects the performance of multimodal data fusion to a large extent, and each modality may suffer from noise interference at different moments. When the data are affected by noise, the results will not be able to accurately represent the original features [61]. In addition, the effectiveness of multimodal fusion will also be significantly impacted if the modalities are not well aligned with each other, as in the case in which data from brain waves and eye tracking are not synchronized. To summarize, researchers in this field should pay attention to addressing the interference of time-variant effects and noise on multimodal data fusion.

#### 7.2.2. Develop Intuitive Devices for Hazard Recognition

As a tool for effective hazard recognition, intuitive devices can not only monitor multimodal physiological data, but also make judgments and give feedback according to the obtained data. To date, intuitive devices have been developed based on two kinds of data: brain activity states (such as EEG) and eye-tracking (such as scan path). The studies of this subject are closely combined with psychology knowledge [62].

The main issue in intuitive device development based on eye-tracking technology is that there is no consensus on the processing of eye-tracking data in the construction industry. Some studies measured workers’ cognitive load or safety attention through cross-sectional visual signal concentration (such as gaze duration and times) [40], whereas others used pupil diameter [63]. Additionally, a small number of studies used time-varying trajectory quantitative analysis as evidence of the mental state [64].

(I) In principle, however, different kinds of information processing can produce the same results. For instance, a portion of studies interpreted the average fixation duration as the degree of concentration and found that the duration of fixation reflects the time of individual understanding and processing, which means that the shorter the time, the less attention, leading to recognition errors [65]. However, partial studies interpreted the same indicator as the experience of subjects; that is, the shorter the fixation time, the less time spent in the scenario, resulting in better recognition performance [66]. Moreover, regarding visual trajectories, some scholars claimed that successful hazard recognizers usually spread their attention within the working environment [67]. On the contrary, certain researchers ignored unnecessary distractions in the environment, which is considered to be the key to successful hazard recognition [68]. Obviously, there is no consensus on the interpretation of eye movement data in the construction industry. Therefore, a single indicator cannot satisfy the demonstration of the process of hazard recognition, and research is required to investigate different indicators to describe the physiological process of hazard recognition.

(II) The development of intuitive devices based on electrophysiological signals faces three main problems.

(i) First, numerous researchers collected EEG signals and calculated the voltage value, frequency, vibration, and other parameters to describe the mental state of the participants, such as fatigue, emotion, motivation, and load. Researchers then demonstrated the correlation among these indicators of the participants and hazard recognition performance [69,70]. However, these studies ignored the complex characteristics of EEG signals: each person’s subtle actions in the process of performing tasks may be related to different EEG signals, so the conclusions are likely to be misunderstood due to the action interference required by the experiment [71]. For example, although researchers adopted a longitudinal experiment design to control the differences between groups, allowing the participants to perform the hazard visual search task in a VR environment, they did not specify the participants’ executive actions (squatting, raising hands, raising head, etc.), and such physical actions would significantly interfere with the interpretation of EEG [72].

(ii) Second, the current research on hazard recognition is only based on the distributed brain activity to reveal behavior [73]. However, according to connectionism theory, human behavior should be on the basis of more complex brain region cooperation, rather than a single brain region in a linear manner [74]. Based on the data of EEG analysis, Wang D. et al. noted that it is possible to recognize and quantify a participant’s brain cognition changes by analyzing short time intervals of the raw EEG signal and the combination of different rhythms [75]. Other scholars suggested that the spectral dynamics of EEG in the posterior brain is strongly related to the decrease in vigilance [76]. In this manner, the construction industry’s research on hazard recognition appears to oversimplify the cognitive mechanism of the brain.

(iii) Third, the existing experiments need to reflect on the impact of the amount of data on the results. With regard to the amount of data, both the number of trials and samples were generally too small [77]. Regarding trial times, the number was mainly about 30, which only met the minimum standards of basic statistics and could not easily demonstrate the validity of the results [31].

(III) In light of the shortcomings of the above two series of studies, cross-validation by connecting the two studies through psychological methods is an important means to advance the research on hazard recognition. In previous studies, participants were either equipped with eye-tracking devices to record oculomotor features or quantitative psychological monitoring technologies, such as electroencephalography (EEG) and near-infrared spectrum instrument (NIRS), were used to measure the mental workload evoked by hazardous environments [78]. Because it is difficult to fully demonstrate the cognitive process of potential hazards using unimodal physiological data, other physiological signals are needed to accurately describe the hazard recognition process. Although these attempts were innovative and meaningful for occupational safety research, they failed to identify the psychological mechanisms of cooperation between different types of cognitive functions, such as brain activity and pupillary responses. Accordingly, future researchers may consider making a contribution in this area. Michail et al. proposed that the study requires cross-validation of multiple physiological signals to describe the psychological process [79]; for instance, revealing the cognitive rules of eye activity demands the comparison with specific brain activities, reflecting the adequacy of hypothesis to be tested [80]. Moreover, psychological experimental paradigms applied to hazard recognition need to be further enriched, and the current paradigms mainly include the oddball paradigm, spatial cueing task paradigm, and visual search task paradigm [81].

#### 7.2.3. Establish Integrated Safety Management Platforms

In the research related to organization management, the safety management platform based on big data is a highly promising direction. Traditional construction safety management relies excessively on regular safety inspections and an individual’s ability to recognize potential hazards. However, because of the complex construction scenarios in the construction industry and the difficulty of unification and standardization, traditional safety management tends to have a low rate of hazard recognition. In consequence, building a safety management platform for the construction industry based on big data technology is a highly significant research topic.

At present, automated construction systems designed in Japan and other countries, such as the ABCS and SMART systems, have certain safety management functions; nonetheless, some shortcomings remain. First, the cost is high. This expense makes it difficult to popularize the approach, and a large number of studies have shown the concern about the lack of funds and the high cost of construction machinery manufacturing. Second, it is hard to ensure the stability and safety of the system. Furthermore, the system, whose monitoring technology still needs to be improved, is not specifically developed for hazard recognition and the hazard recognition rate cannot meet users’ requirements. Finally, the lack of interoperability among various information systems and the strengthening of mutual perception among different devices are also problems that need to be solved [82].

Based on the above deficiencies, we indicate that the system should be improved from both internal and external aspects.

(I) Strengthen the stability of the safety management system itself. In terms of industrial robots, the International Organization for Standardization has issued a series of safety management standards for industrial and collaborative robots, and proposed four types of human–robot interaction safety management: safety-related monitoring stop, hand guidance, speed and separation monitoring, and power and force limitation [83], which differ in key control variables, direct human–robot contact, and simultaneous human-robot motion. However, their common essence is to protect human safety during human–computer interaction. Compared with the manufacturing industry, construction scenarios are complex and dynamic, and the construction process is non-standardized, indicating that HCI safety management methods applicable to the manufacturing industry need to be adapted to the construction industry. For this purpose, researchers can propose HCI safety management methods and principles to meet the needs of the construction industry by referring to those methods applicable to the manufacturing industry.

(II) Improve the ability of safety management systems to recognize potential hazards in the surrounding environment. The improvement of this capability depends largely on the crucial technologies involved in CHR-HCI. Most HCI safety management systems based on different technologies are composed of an environmental monitoring system including sensing elements configured at the mechanical stage and an HCI safety warning system configured at the remote-control stage. At present, research in this field focuses on three areas: wearable technologies, virtual technologies, and image sensing technologies.

(i) HCI safety management based on wearable technology can enhance the realism and accuracy of interaction and control. Wearable devices are usually used for visual-assisted control, haptic-assisted control, and optimizing the human–computer interface of construction machinery in the case of insufficient information [84]. Moreover, the integration of wearable devices and remote operation control platforms can increase the realism of manipulation during human–computer interaction [85].

(ii) HCI safety management based on virtual technology mainly includes virtual reality and augmented reality technologies. Compared with sensors and cameras, virtual reality can visualize the blind area of human vision, improve the depth and safety of HCI, and guide operators to complete their tasks [86]. Augmented reality technology can fuse virtual 3D models and the camera surveillance field of view to show operators the invisible construction environment beyond the camera surveillance. Operators can easily identify the depth information and positioning information of the construction site with a fixed view of the camera [87]. In addition, augmented reality can provide a more immersive experience than virtual reality.

(iii) HCI security management based on image sensing technology can identify entities and relationships between them in the environment to enhance the security of HCI [88]. Deep neural network algorithms are widely used in construction resource detection, action recognition, surveillance, and other environmental perception studies [89]. Existing research mostly centers on entity recognition and lacks the comprehension of the relationships between human and machine entities, which is one of the challenges in the field of computer vision [90].

## 8. Conclusions

In this paper, 274 related papers published between 2000 and 2021 were collected from four databases, and a bibliometric method was adopted to analyze the research progress, development, and future trends in this field.

In general, in this study we undertook basic information analysis, keyword co-occurrence analysis, keyword timeline analysis, and cluster analysis. First, we identified the basic information of relevant studies. Second, we constructed a static network with keywords to describe the interrelationships among concepts. Third, term clustering analysis revealed four modules representing the research areas. Fourth, we conducted a timeline analysis and elucidated the changes in research subjects. Finally, we proposed a research framework and three future directions.

The theoretical contributions of this paper are as follows. First, we established a framework showing the current research area by carefully studying the relevant documents, and provided a systematic summary and overview of the current research status. In addition, we explored the relationship between hazard recognition and human–computer interaction, enriched the theory related to CHR-HCI, identified the current research results and their interrelationships, and then identified more helpful directions for future researchers.

In terms of practical contributions, many conclusions drawn from the bibliometric approach were demonstrated. The introduction of human–computer interaction technology has injected new vitality into the field of construction engineering safety and introduced a series of new research topics. As a result, possible essential research topics for future practical research include “how to make multimodal methods better serve experimental data processing of hazard recognition”, “how to develop intuitive sensors and devices”, and “how to build a safety management platform for human-computer interaction based on big data”.

The limitations of this paper include two main points: one is the limitation of the data. In spite of the broad coverage of the four databases, follow-up research will be undertaken to select documents from more academic databases. The other is the limitation of the method. Future researchers are recommended to use quantitative methods, empirical data, and mathematical models to explore the impact of human–computer interaction on the research progress in the field of construction safety.

## Figures and Tables

**Table 1:** An example of paper retrieval.

Search Attributes	Values Used in the Search
Database	Scopus
Keywords	Construction hazard recognition; Human-computer interaction
Boolean operators	(TITLE-ABS-KEY(construct*) OR TITLE-ABS-KEY(build*) OR TITLE-ABS-KEY(erect)) AND (TITLE-ABS-KEY(hazard*) OR TITLE-ABS-KEY(hazard*) OR TITLE-ABS-KEY(peril) OR TITLE-ABS-KEY(risk*) OR TITLE-ABS-KEY(threat*)) AND (TITLE-ABS-KEY(recogni*) OR TITLE-ABS-KEY(identif*) OR TITLE-ABS-KEY(supervis*) OR TITLE-ABS-KEY(detect*) OR TITLE-ABS-KEY (inspect*) OR TITLE-ABS-KEY(realiz*) OR TITLE-ABS-KEY(cogni*) OR TITLE-ABS-KEY(notice*) OR TITLE-ABS- KEY(perceiv*) OR TITLE-ABS-KEY(verif*)) AND (TITLE-ABS-KEY(computer) OR TITLE-ABS-KEY(machine) OR TITLE-ABS-KEY(robot) OR TITLE-ABS-KEY(sensor)) AND (TITLE-ABS-KEY(collaborat*) OR TITLE-ABS-KEY(cooperat*) OR TITLE-ABS-KEY(interact*) OR TITLE-ABS-KEY(combin*)) AND PUBYEAR > 1999

Note: The “*” means searching the words with the same letters before “*”. For instance, “identif*” means searching the words including “identify”, “identification”, “identified” and so on, i.e., any word starting with the letters “identif”.

**Table 2:** The top three most frequently occurring keywords in each cluster except the cluster label.

Keywords	Occurrence Times	Cluster Label
Accident Prevention	24	Computer Vision
Support Vector Machine	20	Computer Vision
Three-Dimensional Computer Graphics	15	Computer Vision
Risk Perception	20	Ergonomics
Risk Management	13	Ergonomics
Human Resource Management	9	Ergonomics
Machine Learning	31	Computer Simulation
Neural Networks	28	Computer Simulation
Monte Carlo Methods	22	Computer Simulation
Safety Training	14	Virtual Reality
Augmented Reality	13	Virtual Reality
Forecasting	10	Virtual Reality

## Data Availability

The data presented in this study are available on request from the corresponding author.
